# Boosting Photovoltaic Respond Through Molecular Engineering in Organic Manganese (II) Bromide for High‐Sensitivity X‑Ray Detection

**DOI:** 10.1002/advs.202507896

**Published:** 2025-06-10

**Authors:** Feifei Chai, Youkui Xu, Lanlan Li, Guoqiang Peng, Hengxin Li, Guiqiang Li, Dongdong Li, Qian Wang, Zhun Yao

**Affiliations:** ^1^ College of Mechanical and Electrical Engineering Henan Agricultural University Zhengzhou 450002 China; ^2^ School of Physical Science and Technology & Lanzhou Center for Theoretical Physics & Key Laboratory of Theoretical Physics of Gansu Province Lanzhou University Lanzhou 730000 China

**Keywords:** 0D structure, DMA_2_MnBr_4_ and PTA_2_MnBr_4_, metal halide, X‐ray detector

## Abstract

Zero‐dimensinoal (0D) manganese‐based metal halides (MHs) demonstrate excellent structural tunable through organic cation design, enabling precise control of Mn─Mn distances to modulate critical photoelectric physical properties. Here, two novel 0D PTA_2_MnBr_4_ (PTA = phenyltrimethylammonium) are synthesized and DMA_2_MnBr_4_ (DMA = dimethylammonium) wafer with a manipulation of octahedra are synthesized by a precise molecular design. Structural analysis reveals that the PTA‐functional group with extended *π*‐conjugation lengths increases the Mn─Mn distance, results in a lower energy transfer and reduced exciton binding energy compared to DMA⁺ (linear carbon chain). Consequently, PTA_2_MnBr_4_ demonstrates an enhanced photoluminescence quantum yield and prolonged exciton lifetime. The engineered X‐ray detector based on PTA_2_MnBr_4_ wafer exhibits a higher sensitivity of 1122 µC Gy_air_^−1^ cm^−2^ and a lower detection limit of 95 nGy_air_ s^−1^, while DMA_2_MnBr_4_ device are 708.2 µC Gy_air_^−1^ cm^−2^ and 180 nGy_air_ s^−1^, respectively, which paves the way for high‐efficiency photoelectronic applications. This suggests that molecular engineering is a robust approach for designing high‐performance 0D manganese halides for radiation detection.

## Graphical abstract

This work successfully synthesized novel 0D Mn^2^⁺‐based metal halides (MHs) PTA_2_MnBr_4_ and DMA_2_MnBr_4_ wafers by a precise molecular design the distance between Mn atoms. The larger PTA⁺ benzene ring structure reduces energy transfer. Importantly, the X‐ray detector based on PTA_2_MnBr_4_ wafers achieved the high sensitivity of 1122 µC Gy_air_^−1^ cm^−2^, an low LoD of 95 nGy_air_^−1^ s^−1^, and superior stability.

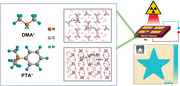

## 1 Introduction

Inorganic semiconductors based X‐ray detectors, such as silicon (Si),^[^ ^1^, ^2^ ^]^ cadmium zinc telluride (Cd(Zn)Te),^[^ ^3^ ^]^ lead iodide (PbI_2_)^[^ ^4^ ^]^ have exhibited a wide range of applications in defense, medical diagnostics, and nondestructive inspection.^[^ ^5^, ^6^ ^]^ However, conventional inorganic semiconductors X‐ray detectors face challenges in meeting the increasing demand for detection, because of their complex preparation process, high cost, and relatively high operating voltage requirements.^[^ ^7^, ^8^, ^9^ ^]^ For instance, Cd(Zn)Te requires high‐temperature (over 1000 °C) and uneconomical preparation by the Czochralski or Bridgman crystal growth method. Furthermore, it can only achieve the required sensitivity at operating voltage exceeding 500 V.^[^ ^10^, ^11^ ^]^ Nowadays, metal halides (MHs) have emerged as a promising candidate for low‐dose X‐ray detection due to its high X‐ray attenuation coefficient (α), large mobility‐lifetime (µτ) product, low cost of raw materials and crystal growth (<0.3 dollar cm^−3^).^[^ ^7^, ^12^, ^13^, ^14^ ^]^ As a result, the MHs X‐ray detectors generally exhibit remarkably sensitivity, low detection limit (LoD), and high signal‐to‐noise ratio (SNR).^[^ ^15^ ^]^ Unfortunately, the majority of organic halide materials are lead‐based compounds, which are harmful to the environment. Additionally, the valence of lead‐free tin‐based materials tends to shift from bivalent to quadrivalence, leading to poor photovoltaic performance.^[^ ^16^, ^17^, ^18^ ^]^ To overcome those limitation, manganese (II) halides have been considered as a promising alternative because of their excellent photoelectric performance and stability.^[^ ^19^, ^20^, ^21^ ^]^ In particular, the precise spatial separation of Mn^2^⁺ polyhedra through tailored organic cation design, driving the formation of specific crystal structures (2D, 1D, and 0D) with exceptional physical and chemical properties.^[^ ^22^, ^23^, ^24^ ^]^ In 2018, Ma et al. reported 0D C_38_H_34_P_2_MnBr_4_ X‐ray scintillators exhibit excellent linear response to X‐ray dose rate and high resolution.^[^ ^25^ ^]^ Recently, Xia et al. demonstrated 0D TPP_2_MnBr_4_ X‐ray scintillators exhibit linear response to X‐ray dose rate and high resolution.^[^ ^26^ ^]^

In the [MnX_4_]^2−^ framework of 0D Mn‐MHs, each Mn^2^⁺ ion is bonded to four halogen ions, forming an isolated tetrahedron that is separated and surrounded by an insulating unit.^[^ ^23^, ^27^ ^]^ Polyomino groups can form more hydrogen bonds with inorganic metal frameworks, which can enhance intermolecular interactions, and thus enabling the formation of unique crystal structure with superior photoelectric physical property such as bandgap and melting point etc.^[^ ^28^, ^29^ ^]^ Therefore, an appropriate design of monovalence amine enables the material to achieve optimized bond length and angle between Mn^2+^ and halogen, thereby alter the arrangement of the surrounding [MnX_4_]^2−^ octahedral. Molecular engineering is an effective approach to design monovalent amine and precisely modulate crystal structural configurations. Compared to traditional techniques like solvothermal synthesis and chemical vapor deposition, molecular engineering operates under ambient reaction conditions, enhancing both process repeatability and product yield. Such atomic‐level precision enables the rational synthesis of manganese halide with predetermined dimensions and morphologies for specific applications. Although the smaller amine size could be shortening Mn─Mn distance, making the crystal structure more robust, it is easy to form energy transfer channel among luminescent centers causes PL quenching.^[^ ^30^, ^31^, ^32^, ^33^ ^]^ While long‐chain organic amines tend to impede charge transfer, their aromatic counterparts with delocalized *π*‐electrons demonstrate effectively modulates electron cloud distribution and thus enhanced electron‐donating capabilities. The long carbon chain with functional benzene ring may offer promising prospects: 1) The extended Mn─Mn distance in the perovskites leading to the excitons more localized is similar to a quantum well, inhibiting the transfer of excitation energy between adjacent Mn^2+^ light‐emitting centers, functionalization for reducing non‐radiative transitions and enhancing photoluminescence (PL) intensity.^[^ ^32^, ^34^, ^35^ ^]^ 2) The *π*‐conjugated spacer cations exhibits reduced exciton binding energy (*E_b_*) and elevated dielectric constant, inducing dielectric mismatch between the organic and inorganic layer, which allows for weakening dielectric confinement effect, modulating electrostatic interaction and promoting charge transport.^[^ ^36^, ^37^, ^38^ ^]^ However, abundant monovalent amine design in Mn‐MHs is focused on theoretical analysis and structure design, experimental research and practical application are lacking.

Herein, we reported two novel 0D DMA_2_MnBr_4_ ((C_2_H_8_N)_2_MnBr_4_) and PTA_2_MnBr_4_ (C_9_H_14_N)_2_MnBr_4_) wafers, featuring distinct differences in their functional organic cation chain. Systematic characterization revealed that the extended Mn─Mn distance in PTA_2_MnBr_4_ induces enhanced steric hindrance, which effectively suppresses energy transfer and reduces non‐radiative recombination. Moreover, the PTA⁺ cation with a benzene ring induce a smaller exciton binding energy compared to DMA⁺, that is correlate with superior optoelectronic performance. As a result, the X‐ray detectors based on PTA_2_MnBr_4_ shows an excellent higher sensitivity (1122 µC Gy_air_^−1^ cm^−2^), and lower detection limit (95 nGy_air_ s^−1^) than DMA_2_MnBr_4_ (708.2 µC Gy_air_^−1^ cm^−2^, 180 nGy_air_ s^−1^). The outstanding performance of this novel 0D organic manganese halides X‐ray detector demonstrates great potential in high‐efficiency photovoltaic and photoelectronic applications.

## 2 Result and Discussion

**Figure 1:**
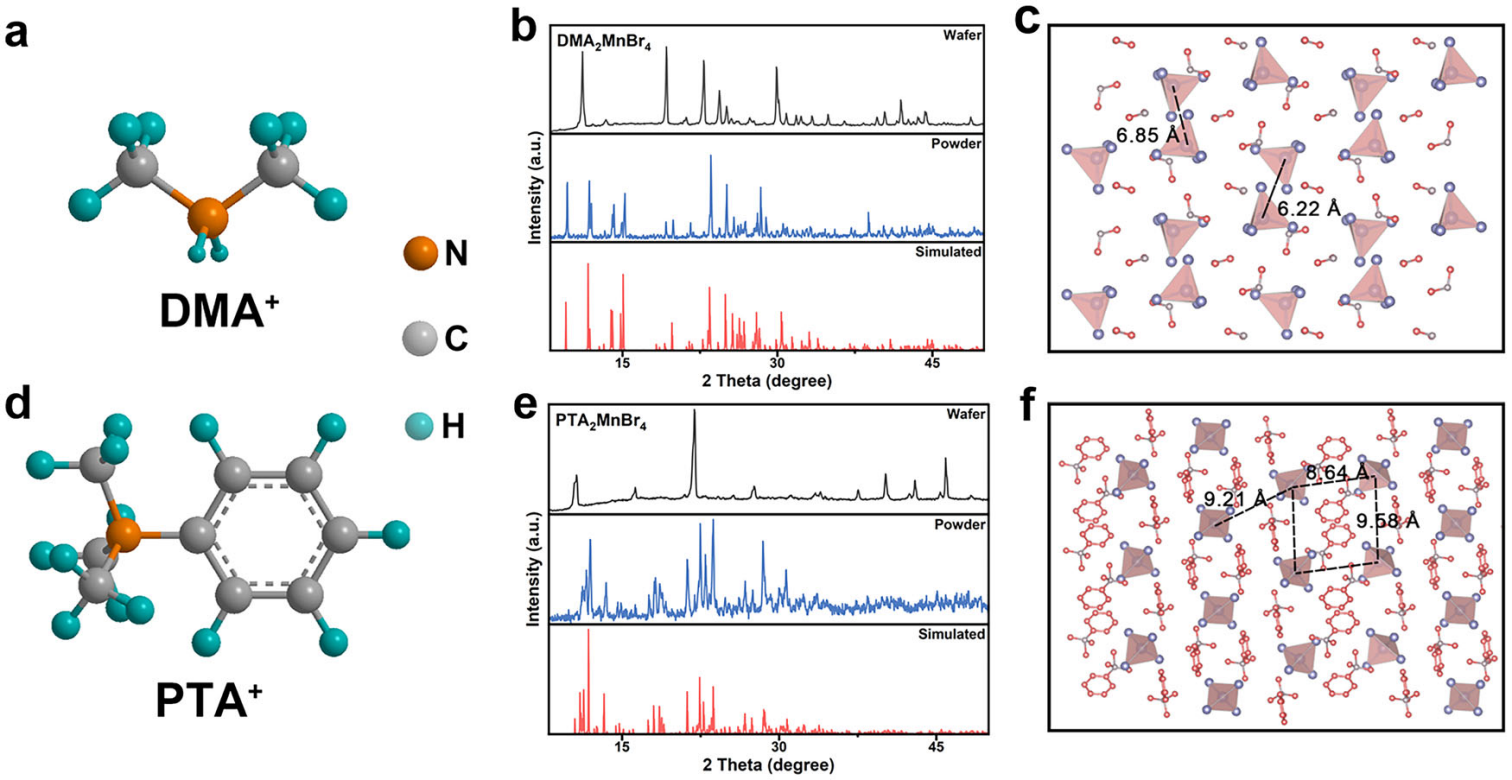
Crystal structure characterization of DMA_2_MnBr_4_ and PTA_2_MnBr_4_ wafers: a,d) The molecular structures of DMA_2_MnBr_4_ and PTA_2_MnBr_4_; b,e) The XRD pattern of DMA_2_MnBr_4_ and PTA_2_MnBr_4_; c) The stacking diagram of DMA_2_MnBr_4_ is projected along a‐axis; f) The stacking diagram of PTA_2_MnBr_4_ is projected along b‐axis.

The molecular structures of DMA_2_MnBr_4_ and PTA_2_MnBr_4_ are illustrated in **Figure** **1**a,d. The PTA^+^ cation incorporates a benzene ring, while DMA^+^ is composed of linear carbon chain cations, which endow it functionality. Both wafer and powders X‐ray diffraction (XRD) of DMA_2_MnBr_4_ and PTA_2_MnBr_4_ were obtained to analyze the crystal structure prepared by melt preparation method. The diffraction peak of DMA_2_MnBr_4_ crystal is matching well with the reported standard XRD cards,^[^ ^39^ ^]^ confirming phase purity without impurities (Figure 1b). As shown in Figure 1c, the crystal structure reveals that each [MnBr_4_]^2−^ tetrahedron consists of a Mn^2+^ site bound to four Br^−^, independently dispersed along the a‐axis direction. Moreover, organic chains fill the space adjacent tetrahedrons, forming a standard 0D structure. Similarly, PTA_2_MnBr_4_ exhibits analogous structural characteristics (Figure 1e) with its diffraction peak is also perfectly matching with the simulated data. Figure 1f illustrates the accumulation diagram of PTA_2_MnBr_4_ projected along the b‐axis, which also confirms its standard 0D structure. From the morphological images of PTA_2_MnBr_4_ and DMA_2_MnBr_4_ in Figure S1 (Supporting Information), we observed that both two compound wafers have good crystallization quality.

The distance between adjacent Mn atoms varies with the dispersion degree of [MnBr_4_]^2‐^ tetrahedral structure, which can be precisely modulated through strategic selection of organic cations. From the crystallographic parameters of two samples are listed in **Table** **1**, both compounds are monoclinic systems. DMA_2_MnBr_4_ belongs to space group of *P*2_1_ */n* with a unit cell of a = 8.209 Å, b = 11.764 Å, c = 15.237 Å, *α* = *γ* = 90°, *β* = 95.147°, *Z* = 4, *V* = 1465.44 Å^3^. PTA_2_MnBr_4_ belongs to space group of *C*2*/c* with a unit cell of a = 16.888 Å, b = 9.064 Å, c = 46.877 Å, *α* = *γ* = 90°, *β* = 92.765°, *Z* = 4, *V* = 7167.03 Å^3^. Notably, the larger unit cell volume of PTA_2_MnBr_4_ correlates with the elongated Mn─Mn distances in DMA_2_MnBr_4_, attributed to the steric effects of the bulkier PTA⁺ cation compared to the smaller DMA**⁺**. Generally, the photoluminescence quantum yield (PLQY) of these compounds proportionally increases as the average adjacent Mn─Mn distance enhances, due to the weakening of energy transfer between Mn─Mn.^[^ ^40^ ^]^ However, when the Mn─Mn distance is too long, the PL intensity will be saturated. Therefore, an appropriate chain length of organic cations is necessary. The Mn─Mn distances of DMA_2_MnBr_4_ and PTA_2_MnBr_4_ are 6.22, 6.85 Å and 8.64, 9.21, and 9.58 Å (Figure 1c,f), respectively. According to theoretical analysis, the increased Mn─Mn interatomic distances generate enhanced steric hindrance effects that reduces energy transfer, which in turn induces a stronger quantum confinement effect, and superior luminescence performance. Benefit from stronger intermolecular interactions between polyomino groups and inorganic metal frameworks greatly reduces the molecular vibration and rotation of [MnBr_4_]^2−^, PTA_2_MnBr_4_ demonstrates a superior PLQY relative to DMA_2_MnBr_4_ (Figure S2, Supporting Information), suggest suppressed non‐radiative recombination and enhanced radiative efficiency.

**Table 1:** The crystal structure parameters of DMA_2_MnBr_4_ and PTA_2_MnBr_4_.

Compound	DMA_2_MnBr_4_	PTA_2_MnBr_4_
Empirical formula	(C_2_H_8_N)_2_MnBr_4_	(C_9_H_14_N)_2_MnBr_4_
Formula weight	466.77 g/mol	1941.01 g/mol
Crystal system	monoclinic	monoclinic
Space group	*P*2_1_ */n*	*C*2*/c*
a/Å	8.209	16.888
b/Å	11.764	9.064
c/Å	15.237	46.877
α/°	90	90
β/°	95.147	92.765
γ/°	90	90
Volume/Å^3^	1465.44	7167.03
Z	4	4
ρ_calc_ (g/cm^3^)	2.116	1.799

**Figure 2:**
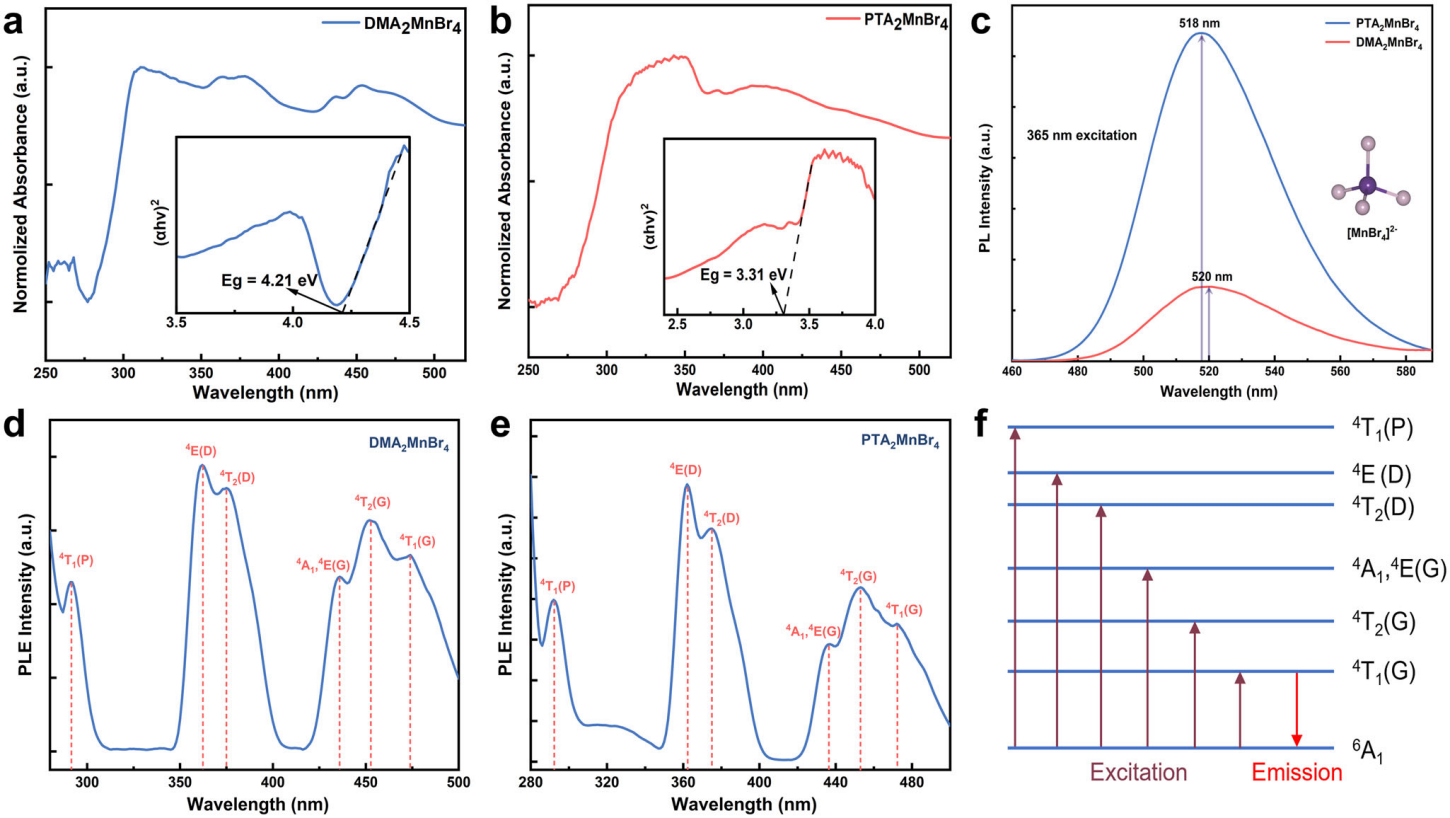
Photophysical properties characterization: a,b) the absorption spectra and the corresponding optical band gaps (illustrations); c) Steady‐state photoluminescence (PL) spectra;d,e) the photoluminescence excitation (PLE) spectra f) the Mn^2+^ electronic transition schematic diagram of DMA_2_MnBr_4_ and PTA_2_MnBr_4_ wafers.

The optical absorption spectra of DMA_2_MnBr_4_ and PTA_2_MnBr_4_ are presented in **Figure** **2**a,b. From the illustrations it can be seen that their band gaps are 3.31 and 4.21 eV, respectively, categorizing both materials as wide‐bandgap semiconductors. A larger bandgap reduces the concentration of hot carriers and lowers the thermal noise of the device and positions them as promising candidates for next‐generation power electronics, optoelectronics, and extreme environment devices. The photoluminescence excitation (PLE) spectra of DMA_2_MnBr_4_ and PTA_2_MnBr_4_ reveal characteristic d‐d transitions of tetrahedrally coordinated Mn^2^⁺ centers.^[^ ^28^, ^41^, ^42^, ^43^ ^]^ In the PL emission spectrum of DMA_2_MnBr_4_ and PTA_2_MnBr_4_ (Figure 2c), a narrow‐band green emission was observed under 365 nm excitation. Specifically, the PL emission of DMA_2_MnBr_4_ had a FWHM of 47 nm at an emission wavelength at 520 nm, while that of PTA_2_MnBr_4_ had a slightly blue‐shifted emission wavelength at 518 nm with a FWHM of 45 nm. Notably, PTA_2_MnBr₄ exhibits 1.8‐fold higher PL intensity compared to DMA_2_MnBr₄, attributed to enhanced quantum confinement effects and steric hindrance from the longer organic cation chain in PTA⁺. As shown in Figure 2d–f, the bands are mainly from three transitions in the two compounds: ^6^A_1_→^4^P, ^6^A_1_ → ^4^D, and ^6^A_1_→^4^G. In DMA_2_MnBr_4_ exhibits six excitation bands at 291, 362, 375, 435, 453, and 474 nm, corresponding to the transitions of ^6^A_1_→^4^T_1_(P), ^6^A_1_→^4^E(D), ^6^A_1_→^4^T_2_(D), ^6^A_1_→^4^A_1_, ^4^E(G), ^6^A_1_→^4^T_2_(G), and ^6^A_1_→^4^T_1_(G), respectively. Similarly, A nearly identical excitation profile is observed for PTA_2_MnBr_4_: 292, 362, 375, 436, 453, 472 nm, corresponding to the transitions of ^6^A_1_→^4^T_1_(P), ^6^A_1_→^4^E(D), ^6^A_1_→^4^T_2_(D), ^6^A_1_→^4^A_1_, ^4^E(G), ^6^A_1_→^4^T_2_(G), and ^6^A_1_→^4^T_1_(G), respectively. The PL emission is derived from ^4^T_1_ to ^6^A_1_ radiative transition of the tetrahedral Mn^2+^ ion within the [MnBr_4_]^2−^ unit. Thus, both DMA_2_MnBr_4_ and PTA_2_MnBr_4_ belong to typical narrow‐band emission of 0D structure.^[^ ^44^ ^]^ This structural feature suppresses non‐radiative recombination pathways through two mechanisms: 1) improved isolation of [MnBr_4_]^2^⁻ units reduce inter‐cluster energy transfer losses, and 2) restricted lattice vibrations decrease electron‐phonon coupling. According to *d*‐ion emission theory, the emission peak position was determined by the strength of the crystal field.^[^ ^45^ ^]^ In this case, the weaker crystal field strength and stronger energy level separation ^4^T_1_‐^6^A_1_ of [MnBr_4_]^2−^ tetrahedra within the DMA_2_MnBr_4_ and PTA_2_MnBr_4_ lattice, leading to a higher energy green emission. These optoelectronic properties suggest PTA_2_MnBr_4_ may improve the efficiency of charge extraction and transmission, while minimizing the loss of photogenerated charges in optoelectronic device applications.

**Figure 3:**
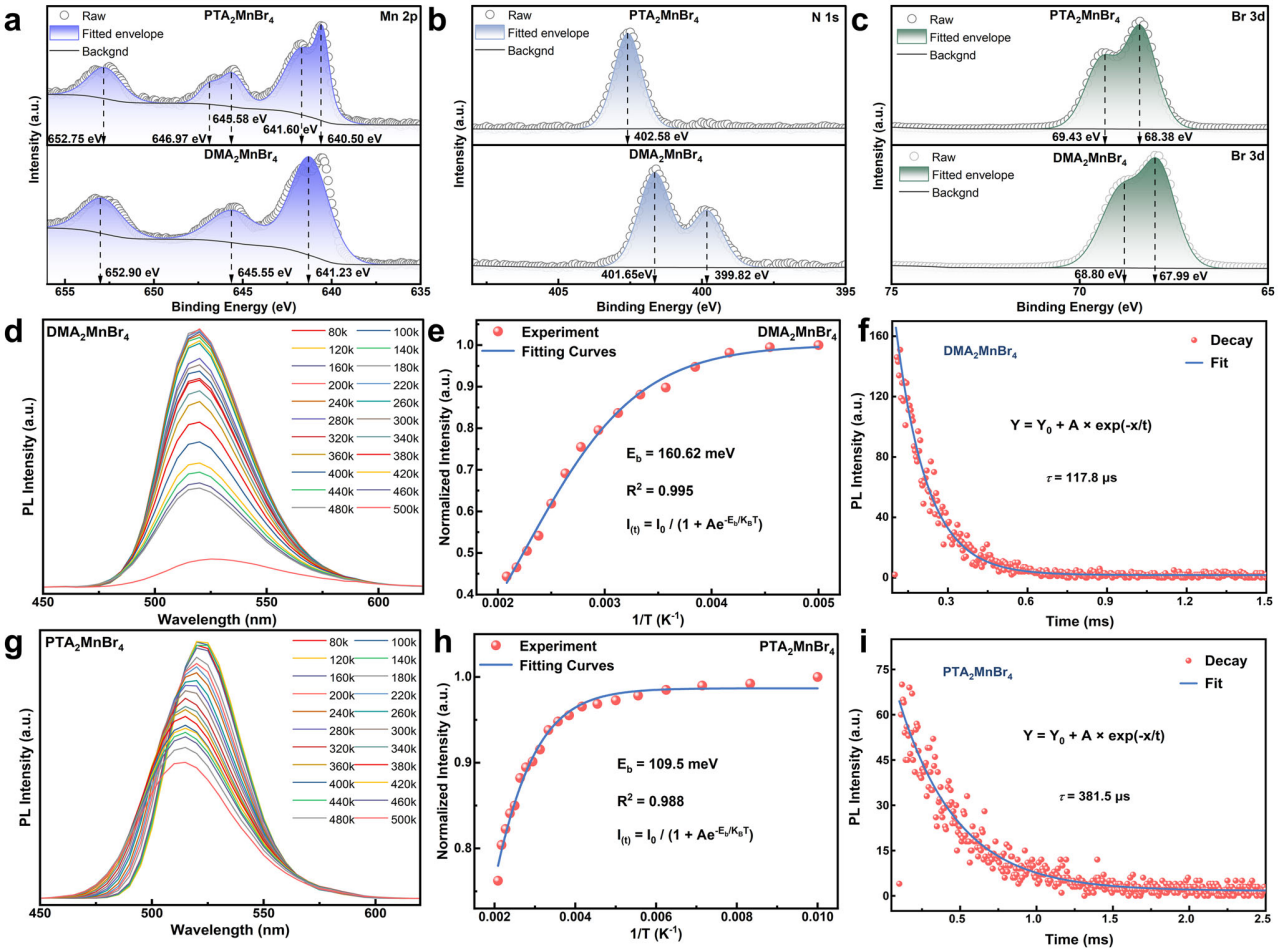
The XPS a) Mn 2p, b) N 1s, c) Br 3d spectra; d,g) the temperature‐dependent PL spectra; e,h) the integrated PL intensity as a function of the inverse temperature; f,i) the TRPL curves of DMA_2_MnBr_4_ and PTA_2_MnBr_4_ wafers.

X‐ray photoelectron spectroscopy (XPS) analysis (**Figure** **3**a–c) reveals distinct electronic environments in DMA_2_MnBr_4_ and PTA_2_MnBr_4_. The binding energies of Br 3d peaks in them shifted from 67.99 to 68.38 eV and from 68.80 to 69.43 eV, respectively. This disparity arises from the steric‐electronic modulation imparted by organic cations: Delocalized π‐electron system of the benzene ring withdraws electron density from adjacent Br⁻ via inductive effects, increasing Br 3d binding energy for hydrogen‐bonded. Figure 3d,g depicts the temperature‐dependent PL spectra of DMA_2_MnBr_4_ and PTA_2_MnBr_4_, respectively. The PL peak of DMA_2_MnBr_4_ exhibits no discernible shift, suggesting a stable bandgap across the temperature range. In contrast, the PL spectrum of PTA_2_MnBr_4_ shows a notable blue shift as the temperature increases, due to thermally activated bandgap narrowing that driven by lattice contraction‐induced enhancement of quantum confinement effects. As shown in Figure 3e,h, by fitting temperature‐dependent PL spectra integrated intensity, the exciton binding energy of DMA_2_MnBr_4_ and PTA_2_MnBr_4_ are calculated to160.62 and 109.5 m eV, respectively. This suggests that PTA⁺ cation with extended *π*‐conjugation lengths has a weaker binding effects in its electronic structure, and facilitates the efficient separation of excitons, which is correlate with superior optoelectronic performance. To investigate exciton lifetime of Mn^2+^ in excited state, we performed time‐resolved PL (TR‐PL) decay measurements of two compounds. As presented in Figure 3f,i, the lifetime *τ* of DMA_2_MnBr_4_ and PTA_2_MnBr_4_ are 117.8 and 381.5 µs, respectively, which are consistent with the value of Mn^2+^ based compounds reported in literature^[^ ^46^, ^47^ ^]^ and further demonstrate the green light emission of two compounds belongs to the d−d transition of Mn^2+^ (^4^T_1_−^6^A_1_). This threefold lifetime enhancement attribute to the different chain length of the organic cation, indicating the lower exciton recombination and non‐radiative recombination in PTA_2_MnBr_4_.

**Figure 4:**
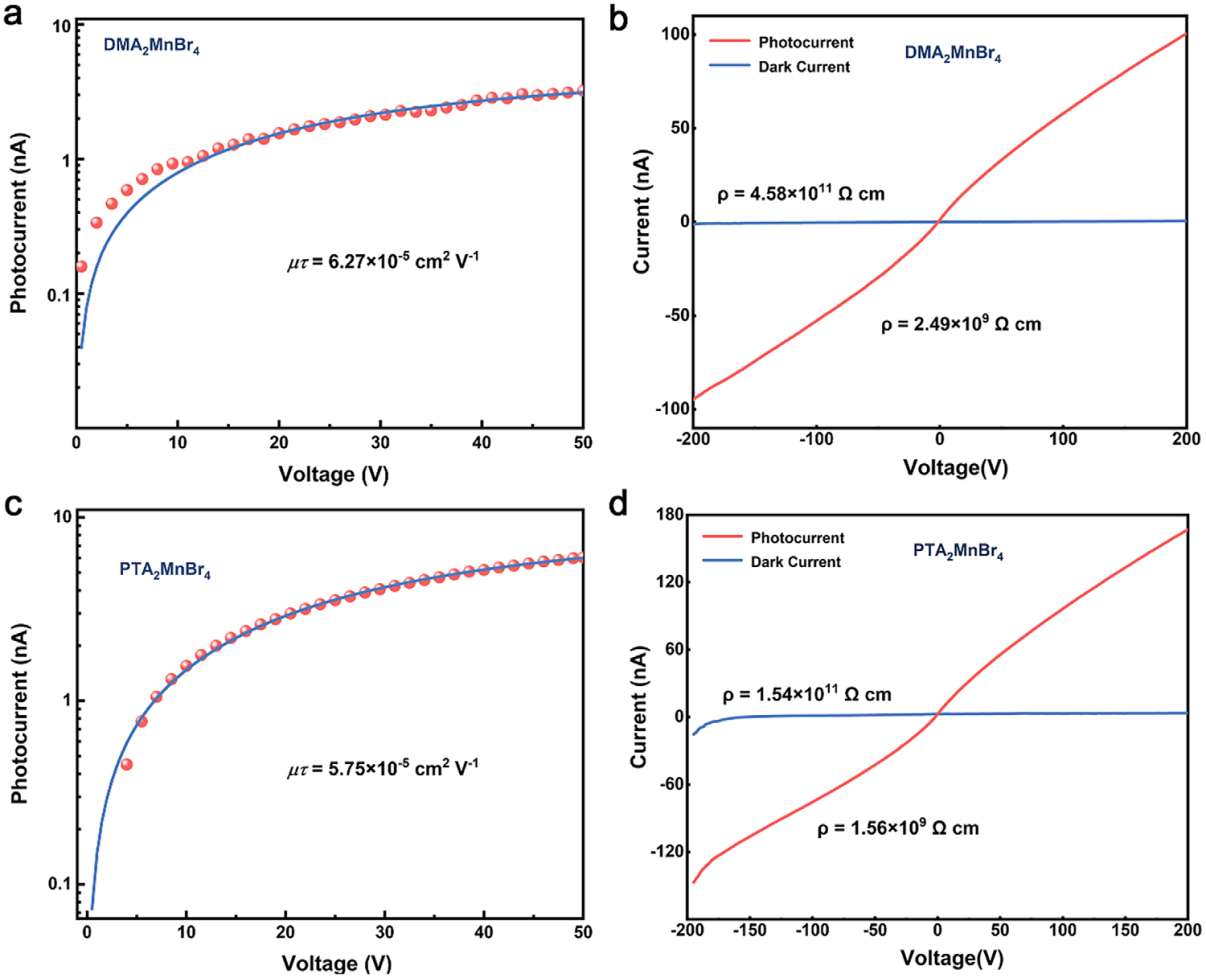
Bias voltage‐dependent photocurrent and current–voltage curves of DMA_2_MnBr_4_ a,c) and PTA_2_MnBr_4_ wafers b,d) under 20 keV X‐ray exposure (200 V bias).

We further study photoelectric properties of DMA_2_MnBr_4_ and PTA_2_MnBr_4_. For photoconductor device, mobility‐lifetime product (*µτ*) is a key parameter to characterize charge transportation. Here, the *µτ* of the DMA_2_MnBr_4_ and PTA_2_MnBr_4_ wafers is calculated by photoconductivity measurement that the induced photocurrent under different bias voltages. As shown in Equation (1),^[^ ^48^, ^49^, ^50^ ^]^ the *µτ* product of single crystal was measured with the modified Hecht Equation (1):

(1) $$I=\frac{\left(I_{0}\mu \tau V\right)}{L^{2}}\;\frac{1-\exp\left(-\frac{L^{2}}{\mu \tau V}\right)}{1+\frac{Ls}{V\mu }}$$

where *I_0_* is saturated photocurrent, *L* is the thickness of wafer, *V* is bias voltages. The *µτ* products of DMA_2_MnBr_4_ and PTA_2_MnBr_4_ wafers are 6.27 × 10^−5^ and 5.75 × 10^−5^ cm^2^ V^−1^, respectively (**Figure** **4**a,c), which is large and almost the same within the fitting error range. As is shown in Figure 4b,d, the resistivity *ρ* of DMA_2_MnBr_4_ and PTA_2_MnBr_4_ wafers are 4.58 × 10^11^ and 1.54 × 10^11^ Ω cm, respectively. Both compounds show a relatively high resistivity *ρ*, which warrants a low dark current of the detector and is expected to be capable of detection at low X‐ray dose rates. All these results demonstrate that DMA_2_MnBr_4_ and PTA_2_MnBr_4_ wafers are promising materials for high‐efficiency X‐ray detection applications.

**Figure 5:**
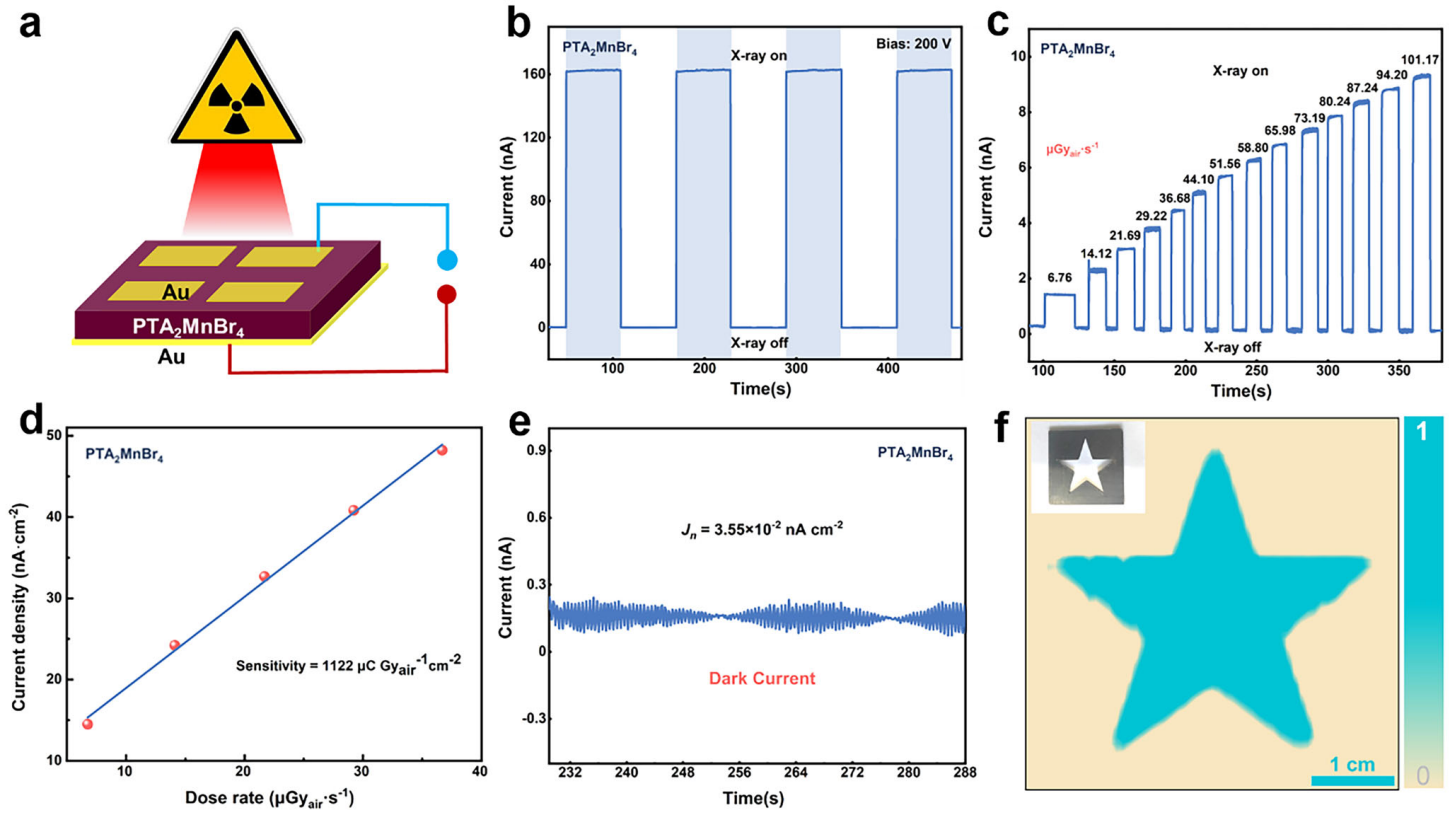
Device performance of PTA_2_MnBr_4_ based X‐ray detectors: a) Schematic diagram of the detector structure; b) X‐ray response characteristics (200 V bias); c) Temporal response of devices to X‐ray source; d) X‐ray photocurrents of PTA_2_MnBr_4_ wafer devices as a function of dose rate; e) Current density of PTA_2_MnBr_4_ wafer device; f) Photograph of a metal key partially wrapped in black plastic and its X‐ray image.

To test this hypothesis, X‐ray detection performance based on PTA_2_MnBr_4_ device was evaluated using a vertical Au/ DMA_2_MnBr_4_ or PTA_2_MnBr_4_ wafer/Au architecture (**Figure** **5**a), where all wafers were synthesized by a low‐temperature melting process to ensure the preparation of dense wafers. The PTA_2_MnBr_4_ device exhibits a low dark current density (*J_d_*) of 2.3 nA cm^−2^ and a high photocurrent density (*J_p_*) of 1804.4 nA cm^−2^, resulting in a high on–off ratio of ≈ 784 (Figure 5b). Figure 5c presents the response characteristics under various X‐ray irradiation doses. As the X‐ray dose rate increases from 6.76 µGy_air_ s^−1^ to 101.2 µGy_air_ s^−1^, the dark current of the device remains stable (0.2 nA), and the photocurrent increases from 1.4 to 9.3 nA. The linear relationship between *J_p_*‐*J_d_* and irradiation dose rate is exhibited in Figure 5d. The sensitivity *S* can be calculated from the slope of net X‐ray response current density–dose rate curves. The *S* of PTA_2_MnBr_4_ wafer was measure as 1122 µC Gy_air_^−1^ cm^−2^, indicating that the direct X‐ray detector based on PTA_2_MnBr_4_ wafer has a relatively large application potential in the field of X‐ray detection. The standard deviation of the current density (*J_n_*) of the device is shown in Figure 5e. According to the calculation formula of signal‐to‐noise ratio (SNR), when SNR is 3, the corresponding irradiation dose is the LoD of the device. Combined with the sensitivity, the LoD of PTA_2_MnBr_4_ wafer detector is identified as 95 nGy_air_ s^−1^. The PTA_2_MnBr_4_ wafer detector was used to capture an image of the pentagram shown in Figure 5f, and the result shows that fine details in the outline of the pentagram can be clearly identified. The performance of X‐ray detector based on direct DMA_2_MnBr_4_ wafer was tested at the same test conditions (200 V bias). From Figure S3a (Supporting Information), the *J_d_* and *J_p_* are 3.5 and 1091.1 nA cm^−2^, respectively, leading to on‐off ratio of 314. The response‐dependent the radiation dose is exhibit in Figure S3b (Supporting Information), as the X‐ray radiation doses increases from 29.83 µGy_air_ s^−1^ to 248.5 µGy_air_ s^−1^, the photocurrent of device increases from 4.5 to 16.5 nA, while the dark current remained stable at 0.4 nA. The *S* value of the DMA_2_MnBr_4_ detector is as high as 708.2 µC Gy_air_^−1^ cm^−2^ (Figure S3c, Supporting Information). Meanwhile, we also performed *J_n_* is calculated by the standard deviation of current density, as shown in Figure S3d (Supporting Information), the LoD of DMA_2_MnBr_4_ wafer detector is identified as 180 nGy_air_ s^−1^.

The performance parameters of detectors based on DMA_2_MnBr_4_ and PTA_2_MnBr_4_ wafers are summarized in **Table** **2**. By comparing these main parameters, it can be found that the dark current density of both compounds remains at a low level, which is conducive to expanding the detection range and reducing noise. The PTA_2_MnBr_4_ device exhibits a lower ion migration and a lower dark current compared to DMA_2_MnBr_4_ wafer, attributed to steric hindrance and quantum constraint effects of the long‐chain PTA. The response photocurrent density of the PTA_2_MnBr_4_ detector is higher than that of DMA_2_MnBr_4_, meaning a lower defect density in PTA_2_MnBr_4_, which is consistent with the previous PL spectrum analysis. The photocurrent density of PTA_2_MnBr_4_ is 1.8 times higher than DMA_2_MnBr_4_, combine with the reduction dark current result in a high response on‐off ratio of PTA_2_MnBr_4_ device, which is more than 2 times better than DMA_2_MnBr_4_. Therefore, the PTA_2_MnBr_4_ wafer X‐ray detector possesses higher sensitivity and lower detection limit under the same electric field intensity and X‐ray irradiation energy. More excitingly, we compared DMA_2_MnBr_4_ and DMA_2_MnBr_4_ in relation to previous manganese‐based device in Table S1 (Supporting Information). Our work has comparable performance, which shows great potential in manganese‐based X‐ray detector.

**Table 2:** Detection performance parameters of devices based on DMA_2_MnBr_4_ and PTA_2_MnBr_4_ wafers.

Material	DMA_2_MnBr_4_	PTA_2_MnBr_4_
*J_d_* (nA/cm^2^)	3.5	2.3
*J*_p_ (nA/cm^2^)	1091.1	1804.4
On‐Off	314	784
*S* (µC Gy_air_^−1^ cm^−2^)	708.2	1122.0
LoD (nGy_air_ s^−1^)	180	95

**Figure 6:**
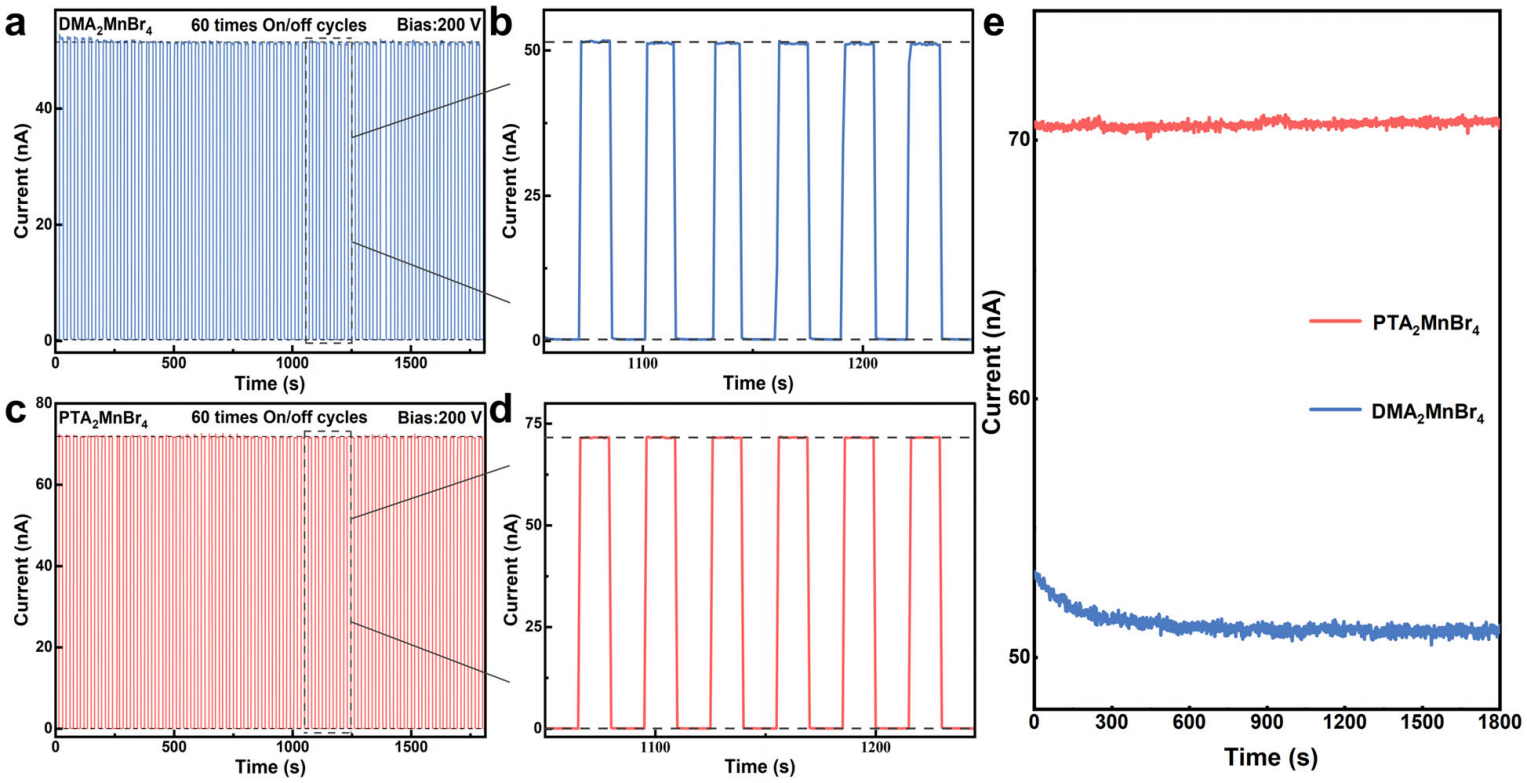
The Stability of DMA_2_MnBr_4_ and PTA_2_MnBr_4_ Based X‐ray Detectors: On/off cycles of a,b) DMA_2_MnBr_4_ and c,d) PTA_2_MnBr_4_ wafer‐based device after 60 consecutive switching cycles; e) The photocurrent stability of DMA_2_MnBr_4_ and PTA_2_MnBr_4_ during 1800 s continuous X‐ray radiation.

The stability of the device fabricated by DMA_2_MnBr_4_ and PTA_2_MnBr_4_ were systematically evaluated through 60 consecutive switching cycles. Remarkably, DMA_2_MnBr_4_ detectors exhibited slight data fluctuations during the test, while the PTA_2_MnBr_4_ X‐ray detectors exhibits good stability with no performance degradation (**Figure** **6**a–d). Long‐term radiation stability was further assessed via continuous X‐ray exposure. As shown in Figure 6e, the PTA_2_MnBr_4_ showed almost constant current after 1800 s of radiation exposure, indicating its exceptional radiation stability. In contrast, the DMA_2_MnBr_4_ exhibited a noticeable decline in photocurrent, suggesting that PTA_2_MnBr_4_ detectors performs better long‐term X‐ray radiation stability.

## 3 Conclusion

In summary, we have successfully synthesized two novel 0D Mn^2+^‐based MHs, PTA_2_MnBr_4_ and DMA_2_MnBr_4_ to systematically study the effect of organic cation chain length on the structural and optoelectronic properties. Those results demonstrate that the extended chain length (PTA_2_MnBr_4_) significantly increase Mn‐Mn distances, result in a lower energy transfer between Mn‐Mn and less non‐radiative recombination compared to DMA_2_MnBr_4_. These structural advantages translate to superior X‐ray detection performance, where PTA_2_MnBr_4_‐based devices achieve a high sensitivity and a low LoD in the range of n Gy_air_ s^−1^, which broadens the selection of materials for high‐performance X‐ray detector. Through a comparative analysis of the two novel synthesized ligands, we have determined that molecular engineering strategy will guide the design of organic cation and enrich the use of X‐ray detectors in commercial manganese halides optoelectronic applications.

## Conflict of Interest

The authors declare no conflict of interest.

## Author Contributions

F.C. and Y.X. contributed equally to this work. F.C. performed data analysis and experimental planning. Y.X conducted the experiments and data analysis. G.Q.L. performed the DFT calculation section. The project was conceived, planned, and supervised by Z.Y., L.L., and Z.J. Some of the experimental tests were supported by H.L., J.L., and X.A. The manuscript was written by Z.Y. and Z.J. All the authors reviewed the manuscript.

## Supporting information



Supporting Information

## Data Availability

The data that support the findings of this study are available from the corresponding author upon reasonable request.
