# Supplementary material for: Boosting Photovoltaic Respond Through Molecular Engineering in Organic Manganese (II) Bromide for High‐Sensitivity X‑Ray Detection
Source: Adv Sci (Weinh). 2025 Jun 10;12(33):e07896. doi: 10.1002/advs.202507896 (PMC12412481; doi:10.1002/advs.202507896)
Supplement: Supplementary file 1 — Supporting Information [file ADVS-12-e07896-s001.docx]

Supporting Information

**Boosting Photovoltaic Respond through Molecular Engineering in Organic Manganese (II) Bromide for High-Sensitivity X‑ray Detection**

*Feifei Chai*^1,#^, *Youkui Xu^2^*^,#^, *Lanlan Li*^1^, *Guoqiang Peng* ^2^, *Hengxin Li ^2^*, *Guiqiang Li^1^*, *Dongdong Li*^1^, *Qian Wang*^2,*^, *Zhun Yao*^1,*^

Dr. F. Chai, Prof. L. Lan, Dr. J. Li, Dr. X. An, Prof. Z. Yao

^1^College of Mechanical and Electrical Engineering, Henan Agricultural University, Zhengzhou, 450002, China.

Dr. Y. Xu, Dr. G. Peng, Dr. H. Li, Prof. Q. Wang

^2^School of Physical Science and Technology & Lanzhou Center for Theoretical Physics & Key Laboratory of Theoretical Physics of Gansu Province, Lanzhou University, Lanzhou 730000, China

E-mail: [qianwang@lzu.edu.cn](mailto:qianwang@lzu.edu.cn); zhunyao@henau.edu.cn

**Keywords:** Metal halide; DMA_2_MnBr_4_ and PTA_2_MnBr_4_; X-ray detector; 0D structure;

**Experimental:**

*Materials preparation*: hydroiodic acid (HBr) and manganese (II) bromide tetrahydrate (MnBr_2_•4H_2_O, 98%) were purchased from Aladdin Reagent Ltd. All the chemicals were used as received without further purification. C_3_H_7_NO(DMF) was purchased from Alfa Company. We confirmed that all the reagents and chemicals were used as received without further purification.

*Synthesis of* dimethyl ammonium bromide C_2_H_8_NBr (DMABr) powder: As shown in **Figure S4**, HBr and dimethylformamide (DMF) were mixed according to a certain volume ratio, and stirred for about 3 h to ensure fully react. Then the mixed liquid is added to the rotary distillation device until it is spun dry into a viscous liquid, and dried at 40°C in a vacuum environment, and finally DMABr powder is obtained. The remaining reagent samples are purchased.

The preparation process of PTABr powder were same to DMABr powder.

*Synthesis of* DMA_2_MnBr_4_ crystal: 0.5040 g DMABr (dimethyl ammonium bromide) and 0.5722 g MnBr_2_•4H_2_O with the stoichiometric ratio of 2:1 were dissolved in 5 mL water. Dry DMA_2_MnBr_4_ crystals were obtained by slowly evaporating the mixture solution at 70 °C in a vacuum oven.

*Synthesis of* PTA_2_MnBr_4_ crystal: the PTABr (Trimethyl phenyl ammonium bromide) and MnBr_2_•4H_2_O (with the stoichiometric ratio of 2:1) were dissolved in water and slowly evaporated to obtain PTA_2_MnBr_4_ crystal.

*Synthesis of* DMA_2_MnBr_4_ wafers: 0.5g of DMA_2_MnBr_4_ powder was placed on clean quartz glass (20 mm×20 mm). The substrate was heated at 175 °C for 10 min to ensure complete melting of DMA_2_MnBr_4_. After that, another clean quartz underwent a preheating treatment at 175 °C on a hotplate and then coated with molten DMA_2_MnBr_4_ for 5min. Finally, the DMA_2_MnBr_4_ wafer was gently remove from quartz splint after cooling to room temperature.

The preparation process of PTA_2_MnBr_4_ wafers were same to DMA_2_MnBr_4_ wafers.

*Device fabrication preparation:* The DMA_2_MnBr_4_ X-ray detector was made by depositing interdigital ~80 nm thickness Au electrodes via vacuum evaporation. The shape of the metal mask is similar to our previous work, and the effective electrode area is 9 mm^2^.

**Characterization Section:**

*Absorbance spectra*: was collected using a FLS920T using the slowest scanning rate

with one-second integration and a 5 nm slit width.

**PL and PLE spectra:** The PL spectra were measured using a PicoQuant FluoTime 300. The PLE spectra results were collected by FLS920T using the slowest scanning rate with one-second integration and a 1 nm slit width.

**XRD:** XRD patterns of the PTA_2_MnBr_4_ and DMA_2_MnBr_4_ wafers were characterized by the Bruker D2 PHASER Diffractometer with the Cu Kα line.

**Time-resolved PL (TR-PL)**：TR-PL was measured using the PicoQuant 300 system with the excitation wavelength of 365 nm.

**PLQY:** PLQY was measured using the C9920-03 integrating sphere.

**Detector performance measurement:** The X-ray detection performance was measured using a copper anode X-ray source with the largest energy at 50 keV and the intensity peak at 20 keV. The dose rate was changed by changing the X-ray tube current and was calibrated with a Radcal ion chamber dosimeter. A Keithley 2635B Source Meter was used to apply the bias voltage and record the response current. For noise current measurement, 1 V bias was exerted onto the device by low noise current amplifier (SR570) and the output was connected to a lock-in amplifier (SR850). SR570 worked in a high bandwidth mode without adding any filter. All measurements were taken at room temperature in the air.

**X-ray imaging Measurement (single-pixel scanning imaging):** The X-ray imaging capability of the detector was measured by moving the objects (the key in the black box) on a home-built x-y scanning system that can collect the current signal of the detector matched with the object positions. Specifically, the object was fixed on an x-y scanning stage and was allowed to move in and out of the X-ray beam in both the x and y directions to obtain a complete image. A Keysight B2902A source meter connected to the x-y scanning system was used to provide a 10 V μm-1 electric field and record the detector current and corresponding position coordinates.

**X-ray imaging Measurement (linear scanning imaging):** The linear scanning array was constructed by 10 individual X-ray detector pixels, for whom each pixel has an effective area of 9 mm^2^. For a typical scanning, objects along one direction with the step size of 3 mm and recording the current response of each pixel at each position to reproduce the image.


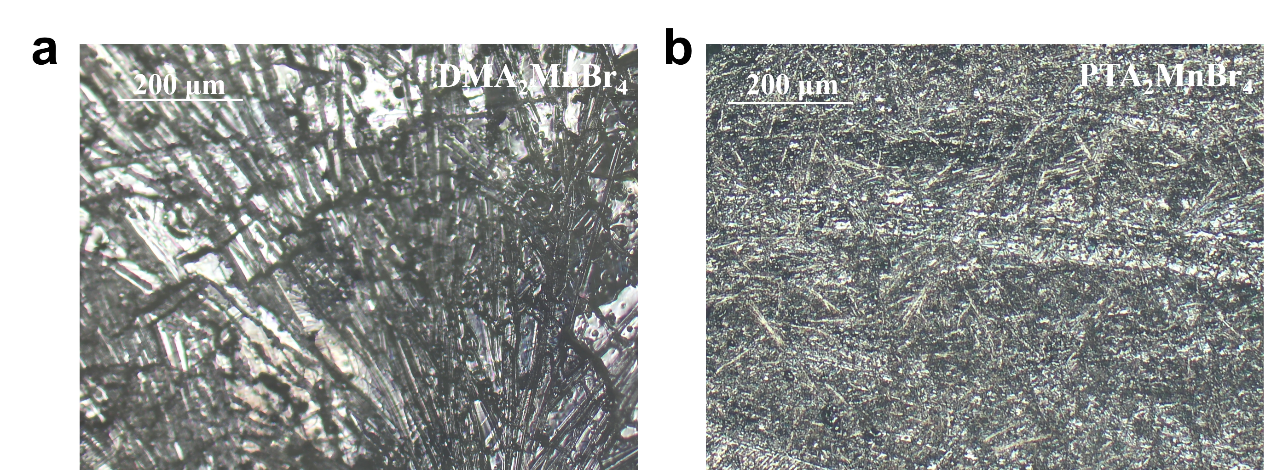


**Figure S1.** The morphology images of (a) DMA_2_MnBr_4_ and (b)PTA_2_MnBr_4_, respectively.

**
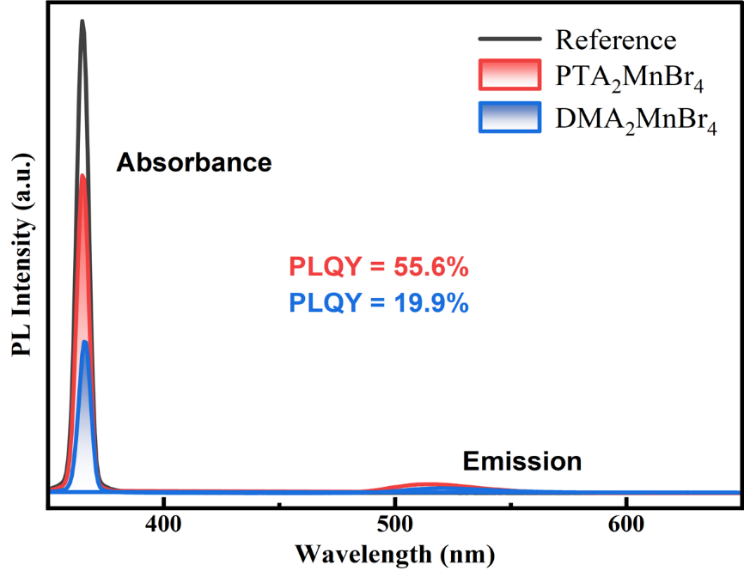
**

**Figure S2.** The PLQY spectrum of PTA_2_MnBr_4_ and DMA_2_MnBr_4_.


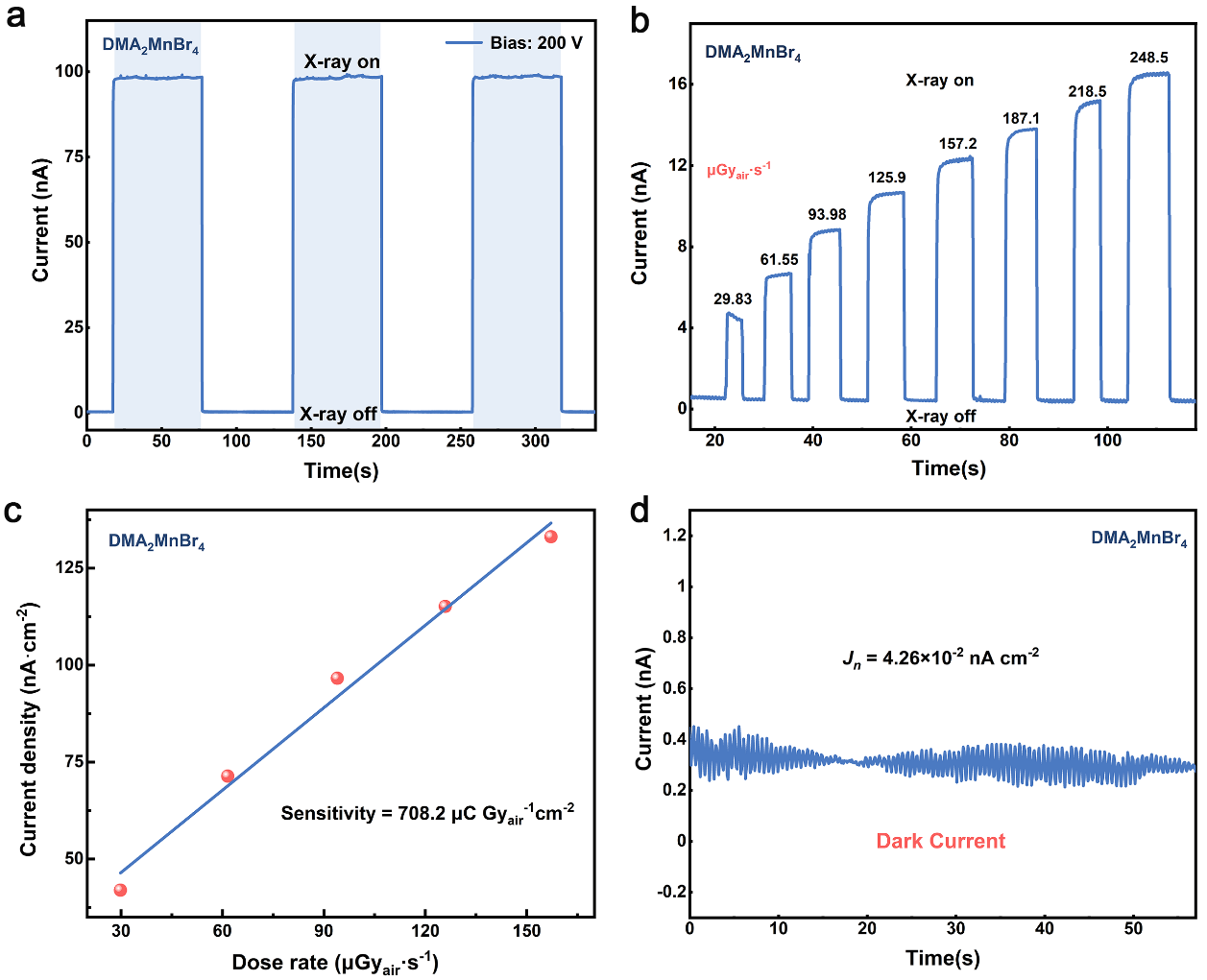


**Figure S3.** Device performance of DMA_2_MnBr_4_ based X-ray detectors (a) X-ray response characteristics (200V bias), (b) Temporal response of devices to X-ray source (c) X-ray photocurrents of DMA_2_MnBr_4_ wafer devices as a function of dose rate. (d) Current density of DMA_2_MnBr_4_ wafer device.


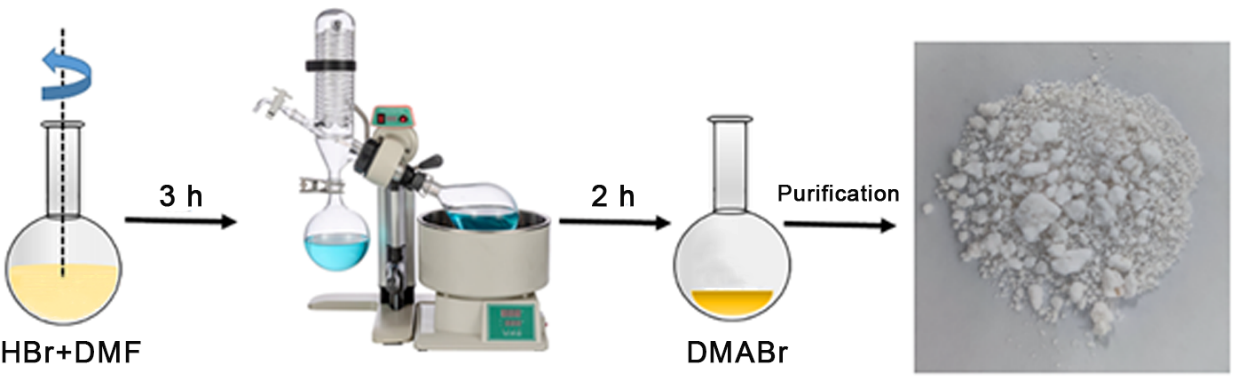


**Figure S4.** Schematic illustration to synthesis of DMABr powder.

**Table S1.** The studies of PTA_2_MnBr_4_ and DMA_2_MnBr_4_ in relation to previous manganese-based X-ray scintillators.

| Performance | *S* (μC Gy_air_^-1^ cm^-2^) | LoD (nGy_air_ s^-1^) | Reference |
| --- | --- | --- | --- |
| PTA_2_MnBr_4_ | 1122.0 | 95 | This work |
| DMA_2_MnBr_4_ | 708.2 | 180 | This work |
| (C_38_H_34_P_2_)MnBr_4_ | / | 72.8 | [1] |
| (BTPP)_2_MnBr_4_ | / | 89.9 | [2] |
| BTP_2_MnBr_4_ | / | 86.2 | [3] |
| (TBA) _2_MnCl_4_ | / | 381 | [4] |
| (ETP) _2_MnBr_4_ | / | 103 | [5] |
| HTP_2_MnBr_4_ | / | 130 | [6] |
| BPP_2_MnBr_4_ | / | <250 | [7] |
| (MTP)_2_MnCl_4_ | / | 144.65 | [8] |
| (PTA)_2_MnCl_4_ | / | 594.06 | [8] |
|  |  |  |  |

**Reference**

[1] L.-J. Xu, X. Lin, Q. He, M. Worku, B. Ma, Nature Communications 2020, 11, 4329.

[2] W. Li, Y. Li, Y. Wang, Z. Zhou, C. Wang, Y. Sun, J. Sheng, J. Xiao, Q. Wang, S. Kurosawa, M. Buryi, D. John, K. Paurová, M. Nikl, X. OuYang, Y. Wu, Laser & Photonics Reviews 2023, 18, 2300860.

[3] W. Shao, G. Zhu, X. Wang, Z. Zhang, H. Lv, W. Deng, X. Zhang, H. Liang, ACS Applied Materials & Interfaces 2023, 15, 932.

[4] S. Cao, C. Li, P. He, J. a. Lai, K. An, M. Zhou, P. Feng, M. Zhou, X. Tang, ACS Applied Optical Materials 2023, 1, 623.

[5] B. Li, Y. Xu, X. Zhang, K. Han, J. Jin, Z. Xia, Advanced Optical Materials 2022, 10, 2102793.

[6] Y. Xu, Z. Li, G. Peng, F. Qiu, Z. Li, Y. Lei, Y. Deng, H. Wang, Z. Liu, Z. Jin, Advanced Optical Materials 2023, 11, 2300216.

[7] S. Wang, H. Chen, Y. Xu, G. Peng, H. Wang, Q. Li, X. Zhou, Z. Li, Q. Wang, Z. Jin, Small 2024, 20, 2403234.

[8] A. A. Karluk, S. Thomas, A. Shkurenko, B. E. Hasanov, J. Mahmood, M. Eddaoudi, C. T. Yavuz, Journal of Materials Chemistry C 2025, 13, 2165.
